# Genetically predicted lipids mediate the association between intrahepatic cholestasis of pregnancy and cardiovascular disease

**DOI:** 10.3389/fcvm.2024.1401010

**Published:** 2024-04-30

**Authors:** Ji Cui, Qilong Zhai, Mengjie Chen, Zhu Yang

**Affiliations:** ^1^ Department of Obstetrics and Gynecology, The Second Affiliated Hospital of Chongqing Medical University, Chongqing, China; ^2^ Department of Hepatobiliary Surgery, The Second Affiliated Hospital of Chongqing Medical University, Chongqing, China

**Keywords:** intrahepatic cholestasis of pregnancy, lipid, cardiovascular disease, Mendelian randomization, GWAS

## Abstract

**Introduction:**

Intrahepatic cholestasis of pregnancy (ICP), the most prevalent liver disorder specific to pregnancy, affects approximately 1.5%-4% of pregnancies. However, the influence of ICP on cardiovascular disease (CVD), including hypertension (HTN) and coronary artery disease (CAD), has not been thoroughly investigated.

**Methods:**

This study explores the causal relationship between ICP and CVD (HTN, CAD) using Mendelian Randomization (MR). Utilizing summary-level data from Genome-Wide Association Studies (GWAS), we applied the inverse-variance weighted (IVW) method, supplemented by sensitivity and reverse MR analyses, to ascertain robustness.

**Results:**

Our findings reveal significant causal links, indicating ICP notably increases the risk of CVD (*P* = 0.001), hypertension (HTN, *P* = 0.024), and coronary artery disease (CAD, *P* = 0.039). A two-step MR analysis highlighted the mediation role of lipid profiles, with LDL, TC, and Apo-B contributing to increased CVD risk by 25.5%, 12.2%, and 21.3%, respectively. Additionally, HTN was identified as a mediator in the ICP-CAD association, accounting for a 14.5% mediation effect.

**Discussion:**

The results underscore the genetic predisposition of ICP to elevate CVD risk and the critical mediating role of lipid levels, emphasizing the need for vigilant lipid monitoring and early intervention in individuals with ICP.

## 1 Introduction

Intrahepatic cholestasis of pregnancy (ICP), the most prevalent liver disorder specific to pregnancy, affects approximately 1.5%–4% of pregnancies (1, 2). The incidence of ICP varies according to geographic location and ethnicity, with a higher incidence in South American populations and a lower incidence in European populations (3). Its clinical manifestations include pruritus and elevated levels of serum bile acids and transaminases (4). Typical symptoms of ICP begin to appear around the third trimester of pregnancy and the condition worsens as the pregnancy progresses. Typically, ICP symptoms subside within 48 h postpartum, and biochemical irregularities normalize within 2–8 weeks (5).

Notably, ICP is linked to several adverse perinatal outcomes, including preterm labor, unexplained stillbirth, and postpartum hemorrhage (6, 7). The pathogenesis of sudden intrauterine death related to ICP remains unclear, but it is hypothesized to involve disruptions in fetal circulation due to abnormal bile acid concentrations. Recent studies of ICP fetal outcomes have shown that the risk of adverse fetal outcomes increases with increasing maternal serum bile acid levels (8). The most effective pharmacologic treatment to improve clinical symptoms and biochemical abnormalities in patients with ICP is Ursodeoxycholic acid (UDCA), and this has also been shown to reduce placental abnormalities and improve placental bile acid transport in *in vitro* studies (9, 10).

The pathogenesis of ICP is multifactorial, involving environmental factors, hormonal changes, and genetic mutations (11). ICP is more likely to occur in winter and in populations in areas with lower dietary selenium intake, suggesting that environmental factors play a role in the development of ICP (12). Reproductive hormones also play a role in ICP, and women with higher levels of estrogen and progesterone are more likely to experience ICP symptoms (13). In terms of genetic factors, the more widely studied gene is ABCB4, and mutations at loci such as ABCB11 and ABCC2 have also been reported in ICP (14).

Associations between ICP and various conditions, such as hepatitis C, non-alcoholic fatty liver disease, cholecystitis, pancreatitis, and autoimmune diseases, have been documented (15). Several studies have shown that ICP may coexist with other pregnancy-related conditions such as pre-eclampsia, acute fatty liver of pregnancy and gestational diabetes (16). Previous studies have shown that ICP patients have a threefold increased risk of gestational diabetes and pre-eclampsia compared to normal pregnant women (17). Both gestational diabetes and pre-eclampsia are recognized as risk factors for cardiovascular disease (18). However, the impact of ICP on cardiovascular disease (CVD) remains underexplored, with most research focusing on fetal cardiac implications (19). Observational studies have indicated an elevated risk of preeclampsia in women with a history of ICP (20). Yet, the influence of ICP on adult cardiovascular event risk, including hypertension (HTN) and coronary artery disease (CAD), requires further investigation. Currently, only a few observational studies have reported on the relationship between ICP and cardiovascular disease, and they have not obtained uniform results. Traditional observational studies in this context are often subject to limitations like residual confounding and reverse causality bias.

Mendelian randomization (MR) refers to an analytical method for assessing causal relationships between observed modifiable exposures or risk factors and clinically relevant outcomes. It provides a valuable tool, especially when randomized controlled trials examining causality are not feasible and when observational studies provide biased associations due to confounding or reverse causality. These issues are addressed by using genetic variants as instrumental variables (IVs) for testing exposure. Because alleles of exposure-related genetic variants are randomly assigned, the results obtained by MR are not affected by confounders and reverse causation (21). Large-scale genome-wide association studies (GWAS) conducted over the past decade have identified many genetic variants associated with cardiometabolic traits and risk factors. These findings have enabled the design of MR, which has been increasingly applied to predict cardiovascular risk factors in recent years. In this study, we used two-sample and two-step MR to explore the relationship between ICP and cardiovascular disease and to elucidate potential mediators in the pathway linking ICP with cardiovascular disease.

## 2 Methods

### 2.1 Study design

**Figure 1:**
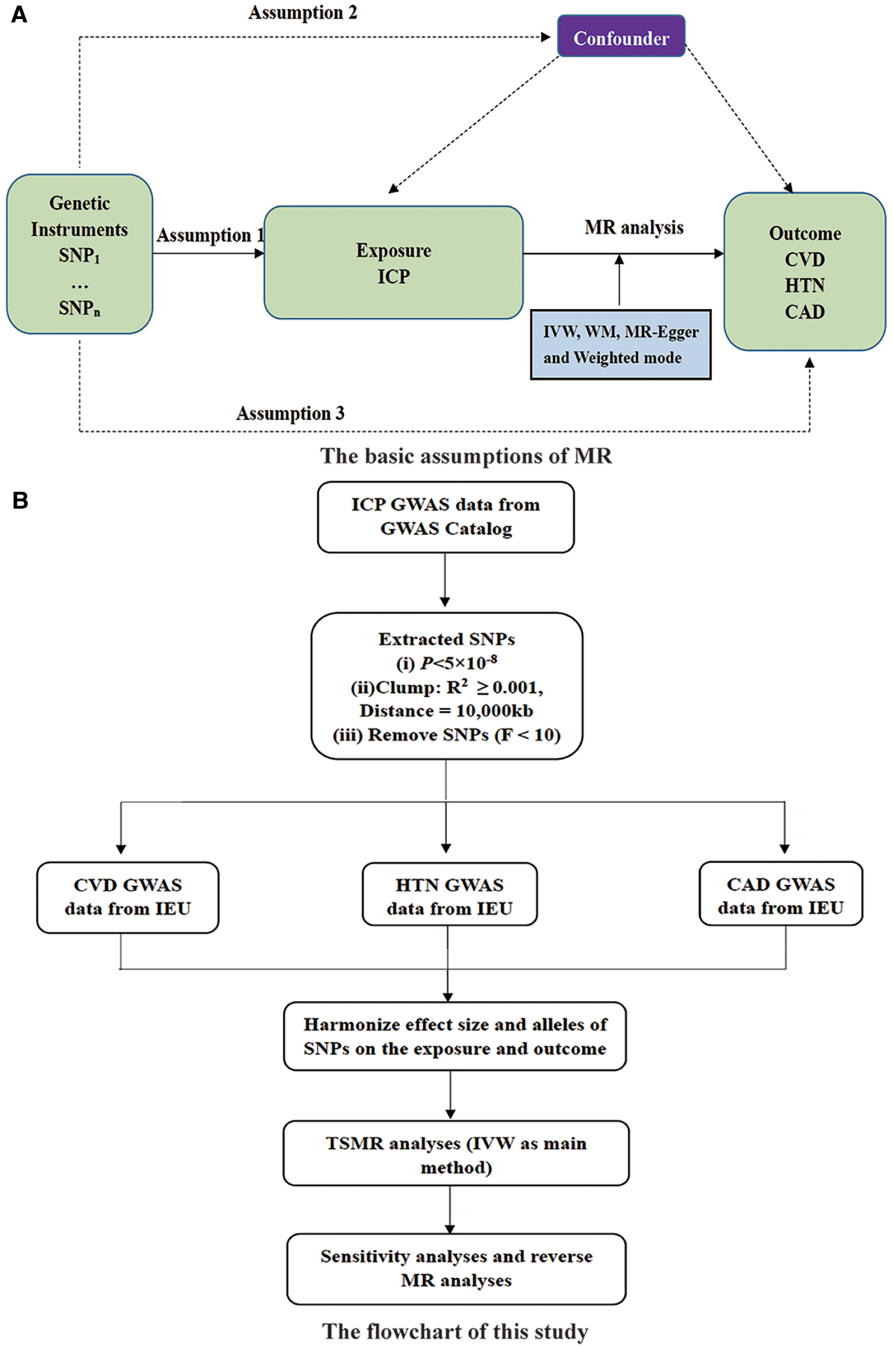
(**A**) The basic assumptions of MR and (**B**) study design. SNP, single nucleotide polymorphisms; ICP, intrahepatic cholestasis of pregnancy; IVW, inverse variance weighted; WM, weighted median; CVD, cardiovascular disease; HTN, hypertension; CAD, coronary artery disease; GWAS, genome-wide association studies; IEU, integrative epidemiology unit open GWAS project.

In this study, we used two-sample MR analysis (TSMR) and two-step MR to investigate the causal associations between ICP and cardiovascular disease, using summary statistics from genome-wide association studies (GWAS). This study adhered to the key principles outlined in the Strengthening the Reporting of Observational Studies in Epidemiology Mendelian randomization (STROBE-MR) guidelines (22). To ensure the accuracy and reliability of the results, this MR study strictly followed the three basic assumptions of Mendelian randomization. First, IVs must be strongly correlated with exposure factors. Second, IVs cannot be directly correlated with the outcome. Finally, IVs must be excluded from being associated with any confounding factors. Figure 1 illustrates the basic assumptions of MR and our study design.

### 2.2 Data sources

The exposure in this MR study was ICP, and the outcome factors were CVD, HTN, and CAD. This study categorizes the potential mediator factors into two major groups, as follows: (1) Blood lipids: low-density lipoprotein [LDL], high-density lipoprotein [HDL], total cholesterol [TC], triglycerides [TG], apolipoprotein A [Apo-A], and apolipoprotein B [Apo-B]; (2) Liver function markers: alanine aminotransferase [ALT], aspartate aminotransferase [AST], alkaline phosphatase [ALP], gamma-glutamyl transferase [γ-GGT], and bile acid. The GWAS summary-level data for ICP were derived from a study by Dixon PH et al. through GWAS Catalog (https://www.ebi.ac.uk/gwas/), which conducted GWAS and a meta-analysis across three studies on ICP, encompassing a total of 1,138 cases and 153,642 controls (23). The GWAS summary-level data about CVD, HTN, CAD, blood lipids and liver function markers used in this study was issued by the Integrative Epidemiology Unit (IEU) open GWAS project (https://gwas.mrcieu.ac.uk/). This project, supported by the Medical Research Council Integrative Epidemiology Unit (MRC IEU) at the University of Bristol, collated and analyzed GWAS data from UK Biobank (24), published articles, and FinnGen biobank (25). There is no significant sample overlap between the GWAS data. More information about the GWAS summary-level data used in this study is presented in Table 1.

**Table 1:** GWAS summary-level data used in this study.

Phenotype	Consortium	Participants	Case	Control	Ancestry	ID
ICP	GWAS Catalog	154,780	1,138	153,642	European	GCST90095084
CVD	IEU	484,598	177,923	306,675	European	ebi-a-GCST90038595
HTN	IEU	462,933	119,731	343,202	European	ukb-b-14057
CAD	IEU	194,427	63,746	130,681	European	ieu-a-9
LDL	IEU	115,082	/	/	European	ebi-a-GCST90092883
HDL	IEU	115,082	/	/	European	ebi-a-GCST90092822
TC	IEU	115,082	/	/	European	ebi-a-GCST90092985
TG	IEU	115,082	/	/	European	ebi-a-GCST90092992
Apo-A	IEU	115,082	/	/	European	ebi-a-GCST90092808
Apo-B	IEU	115,082	/	/	European	ebi-a-GCST90092809
ALT	IEU	150,545	/	/	European	ebi-a-GCST90018723
AST	IEU	150,068	/	/	European	ebi-a-GCST90018724
ALP	IEU	118,886	/	/	European	ebi-a-GCST90018722
γ-GGT	IEU	420,390	/	/	European	ukb-d-30730_irnt
Bile acid	IEU	13,814	/	/	European	ebi-a-GCST90060135

ICP, intrahepatic cholestasis of pregnancy; CVD, cardiovascular disease; HTN, hypertension; CAD, coronary artery heart disease; LDL, low-density lipoprotein; HDL, high-density lipoprotein; TC, total cholesterol; TG, triglycerides; Apo-A, apolipoprotein A; Apo-B, apolipoprotein B; ALT, alanine aminotransferase; AST, aspartate aminotransferase; ALP, alkaline phosphatase; γ-GGT, gamma-glutamyl transferase.

### 2.3 The selection of IVs

In MR analyses, single nucleotide polymorphisms (SNPs) were used as instrumental variables to represent exposures and outcomes to explore causal relationships between them. We screened for SNPs that were strongly associated with exposure at a genome-wide significance level (*P* < 5 × 10^−8^). A total of 11 SNPs were associated with ICP at the genome-wide significant threshold. All of them were not in linkage disequilibrium (LD, *R*^2 ^≥ 0.001 and within 10 mb) and not overlapped with the known risk of CVD. Furthermore, to assess the strength of the screened IVs, this study introduced the F statistic to reflect the ability of the IVs to represent the phenotype. The F statistic was calculated from the sample size, the number of IVs, the minor allele frequency (MAF), and the β-value (26). IVs with F-statistics <10 were regarded as weak genetic instruments. In this study, SNPs with F statistic less than 10 should be removed (27). The details of instrumental SNPs in this study are shown in Table 2. All the F-statistics in this study are greater than 10, indicating that the IVs used satisfy the requirement of a strong association with exposures.

**Table 2:** The details of instrumental SNPs in this study.

Chr	Pos	SNP	Effect allele	Other allele	Eaf	Beta	Se	Pval
14	94378610	rs28929474	T	C	0.020	1.958	0.176	1.041E-28
17	79732281	rs34491636	A	G	0.774	−0.308	0.052	2.620E-09
19	35552195	rs2251250	T	C	0.342	0.290	0.045	1.414E-10
19	47867143	rs296384	T	G	0.844	0.378	0.060	2.501E-10
2	169077231	rs1862069	A	G	0.555	−0.340	0.043	2.043E-15
2	27508073	rs1260326	T	C	0.369	−0.360	0.045	6.838E-16
2	43844604	rs4148211	A	G	0.574	0.529	0.044	1.722E-33
20	44413724	rs1800961	T	C	0.040	0.724	0.115	3.420E-10
4	76490987	rs13146355	A	G	0.454	0.242	0.043	1.995E-08
7	87497783	rs55747905	T	C	0.828	0.485	0.058	7.913E-17
8	58480178	rs10107182	T	C	0.636	−0.403	0.045	1.952E-19

Chr, chromosome; Pos, position; SNP, single nucleotide polymorphism; Eaf, effect allele frequency; Se, standard error; Pval, *P*-value.

### 2.4 Statistical analysis

In this study, we utilized four MR methods—Inverse Variance Weighted (IVW), Weighted Median (WM), MR-Egger, and Weighted Mode—to assess the causal impact of ICP on CVD. Each method is based on distinct assumptions regarding horizontal pleiotropy. Primarily, the IVW approach synthesizes Wald ratios (the ratio of the SNP-associated outcome effect to the SNP-associated exposure effect) through meta-analysis to deduce the aggregate causal relationship between the exposure and the outcome (28). To mitigate reverse causation, we conducted a reverse MR analysis, which swaps the roles of exposure and outcome. The IVW model's heterogeneity was evaluated using Cochran's *Q* test, with a *p*-value less than 0.05 signifying heterogeneity (29). Furthermore, the Mendelian Randomization Pleiotropy Residual Sum and Outlier (MR-PRESSO) test was applied to ascertain the degree of horizontal pleiotropy among the IVs (30). A leave-one-out analysis was also conducted to determine the influence of individual SNPs on the overall results, leading to the immediate exclusion of any identified outliers. Subsequent analyses were carried out post-outlier removal.

**Figure 2:**
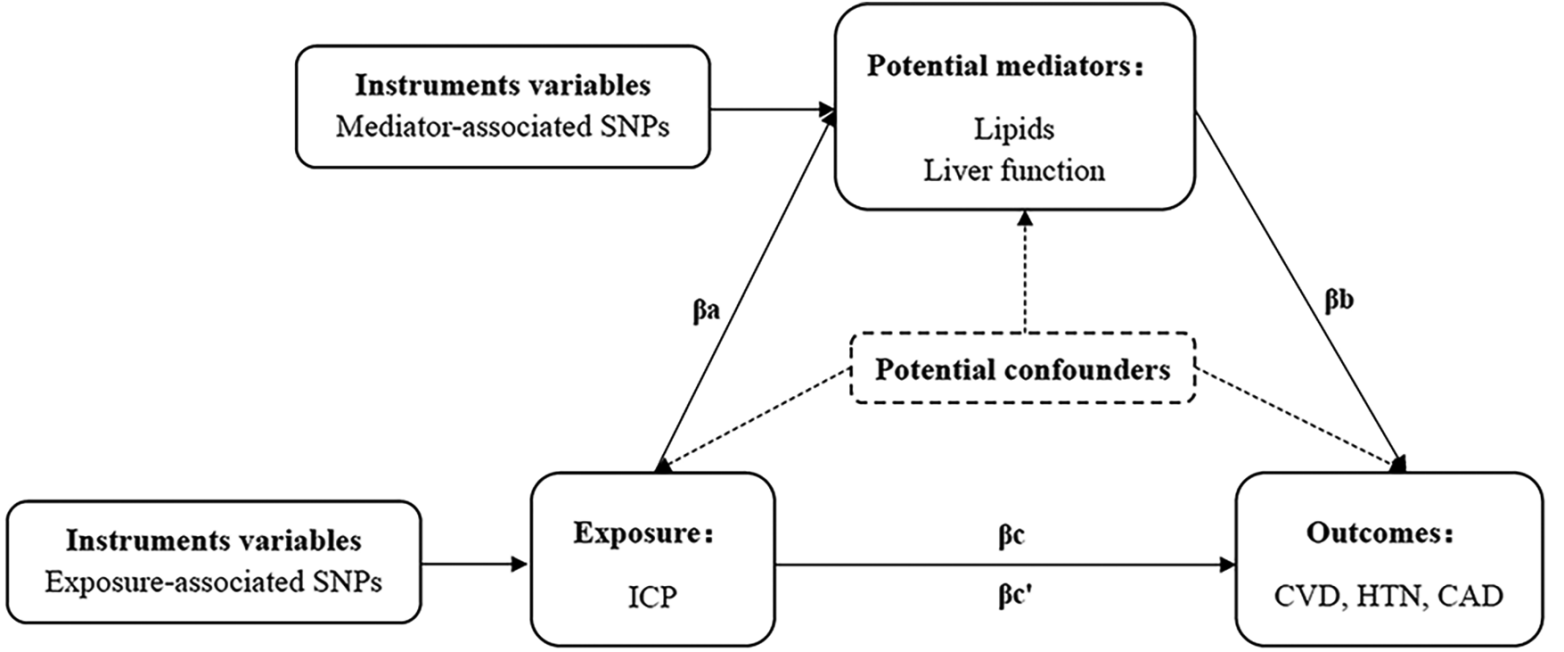
The principle of two-step MR analysis. SNP, single nucleotide polymorphisms; ICP, intrahepatic cholestasis of pregnancy; CVD, cardiovascular disease; HTN, hypertension; CAD, coronary artery disease.

A two-step MR analysis was employed to investigate potential mediation effects by lipid profiles (LDL, HDL, TC, TG, Apo-A and Apo-B) and liver function markers (ALT, AST, ALP, γ-GGT, and bile acid) in the relationship between ICP and VCD. The principle of two-step MR analysis was shown in Figure 2. In the two-step MR analysis, βa is the effect of ICP on the mediator and βb is the effect of the mediator on CVD. βc denotes the total effect (TE) of ICP on CVD and βc’ denotes the direct effect (DE) of ICP on CVD (βc’ = βc—βa*βb). We estimated the proportion of individual mediation by dividing the individual mediated effect (IE) (βa*βb) by the total effect (βc) (31). The indirect effects of ICP on VCD through mediators were quantified using the product of coefficients and the multivariate delta methods. All analyses were performed in R software (version 4.2.0) using the Two Sample MR and MVMR package. In this study, *P* < 0.05 was considered to have a causal relationship. MR estimates were shown as odds ratios (OR) with 95% confidence intervals (CI) or *β* estimates with 95% CI.

## 3 Results

### 3.1 Causal effect between ICP and CVD (CAD, HTN) via TSMR

**Figure 3:**
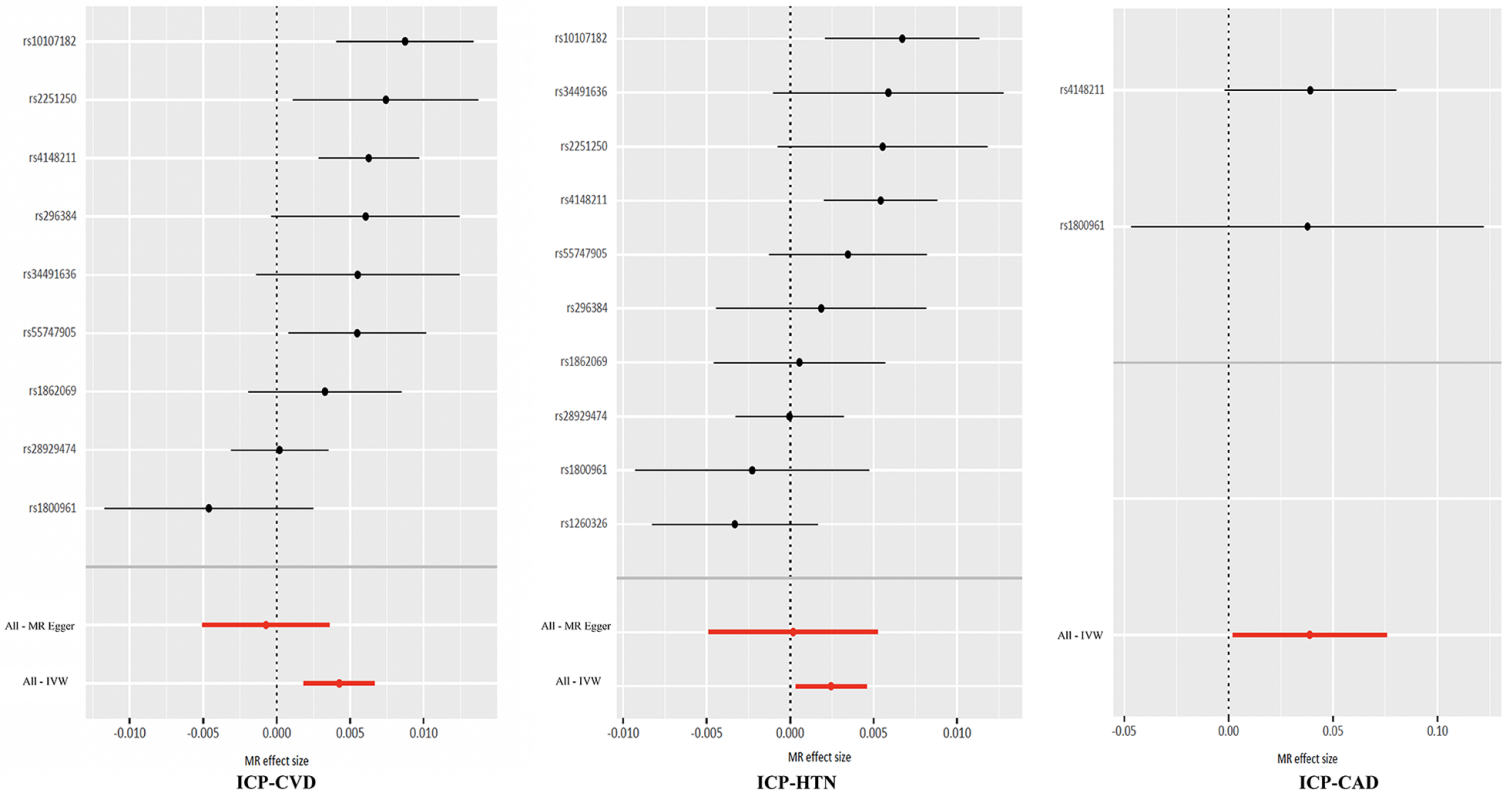
Three causal associations between ICP and CVD, HTN and CAD. ICP, intrahepatic cholestasis of pregnancy; CVD, cardiovascular disease; HTN, hypertension; CAD, coronary artery disease.

In this investigation, three causal associations were established using the IVW method (*P* < 0.05, Figure 3). It was determined that ICP elevates the risk of CVD, as evidenced by an Odds Ratio (OR) of 1.004 and a 95% CI ranging from 1.002 to 1.007 (*P* = 0.001). Comparable causal relationships were observed in the WM analysis, yielding an OR of 1.005 with a 95% CI from 1.003–1.008 (*P* = 7.760 × 10^−6^). Furthermore, a significant correlation between ICP and an augmented risk of HTN was identified through the IVW method, with an OR of 1.002 and a 95% CI between 1.000 and 1.005 (*P* = 0.024). Consistent risk estimates were also derived from MR-Egger, WM, and Weighted Mode analyses. Additionally, a notable link between ICP and CAD was detected via IVW analysis, indicated by an OR of 1.039 and a 95% CI from 1.002 to 1.079 (*P* = 0.039). Reverse MR analysis revealed that CVD, CAD, and HTN do not influence the likelihood of developing ICP. Further details of MR analyses and reverse MR analyses are presented in Supplementary Material Table S1 and S2.

**Figure 4:**
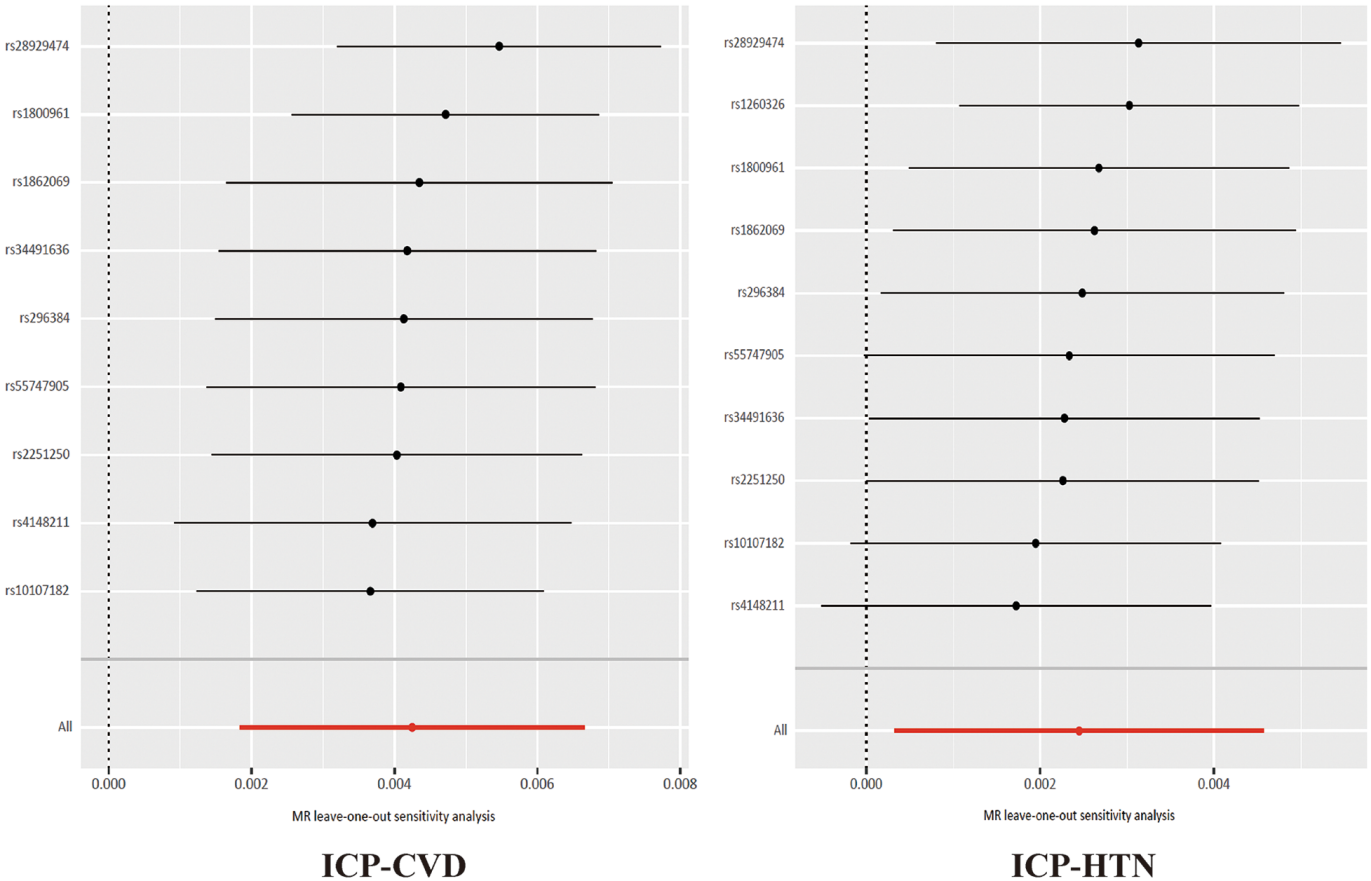
The results of the leave-one-out analyses about ICP, CVD and HTN. ICP, intrahepatic cholestasis of pregnancy; CVD, cardiovascular disease; HTN, hypertension.

After removing outlier SNP, the causal relationship between ICP and CVD still remained. The robustness of the results after removing outlier SNP was assessed through Cochran's *Q* test, the MR-Egger intercept test and MR-PRESSO, as detailed in Supplementary Material Table S3. The MR-Egger intercept tests yielded *P*-values greater than 0.05, indicating an absence of horizontal pleiotropy. Moreover, the lack of detected pleiotropy by the Egger intercept implies that the MR estimates remained unbiased by pleiotropy, notwithstanding observed heterogeneity. The results of the leave-one-out analyses are depicted in Figure 4.

### 3.2 Causal effects of ICP, lipid traits, liver function, and CVD (CAD, HTN) via two-step MR mediation analysis

To investigate the potential mediation of six lipid profiles (LDL, HDL, TC, TG, Apo-A, Apo-B) and five liver function indices (ALT, AST, ALP, γ-GGT, Bile acid) in the relationship between ICP and CVD, CAD, and HTN, a two-step MR analysis was employed (Figure 2). This analysis identified lipids, notably LDL, TC, and Apo-B, as significant mediators in the impact of ICP on CVD, CAD, and HTN (*P* < 0.05). Detailed results of the screening of mediators are shown in Supplementary Material Table S4. However, the liver function indices did not exhibit a substantial mediating effect on the association between ICP and CVD (*P* > 0.05). Specifically, the analysis demonstrated that ICP indirectly increased the risk of CVD by elevating levels of LDL, TC, and Apo-B, with their respective mediation percentages (IE/TE) being 25.5%, 12.2%, and 21.3%. Further exploration into the mediation effect between ICP and CAD indicated that LDL alone contributed a 23.7% mediation effect, while TC and Apo-B showed no significant mediation. Moreover, LDL and Apo-B were found to mediate the relationship between ICP and HTN, with mediation effects of 11.0% and 9.3%, respectively, but no mediating role for TC was observed. Additionally, the analysis extended to the interplay between ICP, CAD, and HTN, revealing that HTN could act as a mediator in the increased risk of CAD attributable to ICP, with a mediation effect of 14.5%. Details of the two-step MR analysis were provided in Table 3.

**Table 3:** The result of two-step MR analysis.

Exposure	Mediator	Outcome	IE (βa*βb)	DE (βc’)	TE (βc)	IE/TE	Pval
ICP	LDL	CVD	0.001	0.003	0.004	0.255	2.952E-04
ICP	TC	CVD	0.001	0.004	0.004	0.122	0.007
ICP	Apo-B	CVD	0.001	0.003	0.004	0.213	0.004
ICP	LDL	HTN	2.682E-04	0.002	0.002	0.110	0.012
ICP	TC	HTN	9.914E-05	0.002	0.002	0.040	0.210
ICP	Apo-B	HTN	2.274E-04	0.002	0.002	0.093	0.031
ICP	LDL	CAD	0.009	0.030	0.039	0.237	0.039
ICP	TC	CAD	0.004	0.035	0.039	0.100	0.300
ICP	Apo-B	CAD	0.006	0.033	0.039	0.163	0.075
ICP	HTN	CAD	0.006	0.033	0.039	0.145	0.032

IE, intermediary effect; DE, direct effect; TE, total effect; ICP, intrahepatic cholestasis of pregnancy; CVD, cardiovascular disease; HTN, hypertension; CAD, coronary artery heart disease; LDL, low-density lipoprotein; TC, total cholesterol; Apo-B, apolipoprotein B.

In conclusion, our findings delineate two primary mediating pathways linking ICP with CVD: Firstly, ICP exerts an indirect causal influence on the risk of CVD, including CAD and HTN, by modulating the concentrations of LDL, TC, and Apo-B. Secondly, ICP indirectly impacts the risk of CAD through its effect on the likelihood of developing HTN.

## 4 Discussion

While the causal link between ICP and CVD remains ambiguous, our MR analysis supports a causal association of ICP with an enhanced risk of CVD, corroborating the observational findings of Shemer et al. (32) Their study reported a marginally increased risk of CVD in later life stages among women with ICP [Hazard Ratio (HR) 1.12, 95% CI 1.06–1.19]. Conversely, the research conducted by Suvi-Tuulia Hämäläinen et al. indicated a lower incidence of CVD-related mortality in women with ICP compared to a control group, a variance potentially attributed to factors such as age at enrollment and follow-up duration (33). Additionally, this analysis extended to explore the causal connections of ICP with CAD and HTN, suggesting that ICP elevates the risks of both conditions. While studies focusing on the ICP-CAD nexus are scant, limited cohort research has hinted at a divergent risk profile for CAD in women with a history of ICP compared to those without. Furthermore, it is widely acknowledged that ICP predisposes to hypertensive complications during pregnancy, and our findings affirm that this risk persists into later life. This study is the first research to find a causal relationship between ICP and CVD at the genetic level using MR methods, which is important for deepening the understanding of ICP complications and guiding follow-up protocols.

Factors such as lipid dysregulation, insulin resistance, endothelial damage, and an enhanced systemic inflammatory response may contribute to the increased CVD risk associated with ICP (34). This study focused on the role of dyslipidemia in abnormal pathophysiologic processes. Research by Chen Y et al. demonstrated a higher prevalence of hyperlipidemia in individuals with ICP compared to those without (5.96% vs. 3.80%), identifying ICP as an independent risk factor for dyslipidemia (35). Building on this, our study employed mediator MR analysis to substantiate the significant indirect effect of ICP on CVD via lipid levels. This mediational MR analysis revealed that LDL, TC, and Apo-B predominantly mediate the indirect impact of ICP on CVD, aligning with the meta-analysis findings of Zhan Y et al. Zhang's research further indicated that severe maternal dyslipidemia was more common in the severe ICP cohort, hinting at a potential link between the severity of ICP and dyslipidemia (36). LDL plays a key role in the development of atherosclerosis (37). LDL is converted from very low-density lipoprotein (VLDL). LDL particles contain about 50% cholesterol and are the most cholesterol-rich lipoproteins in the blood, so they are called cholesterol-rich lipoproteins (38). 95% or more of the Apo in LDL is Apo-B (39). LDL carries cholesterol to peripheral tissues, and most of it is metabolized through the catabolism of the LDL receptor (LDLR) in the hepatocytes and extrahepatic tissues. The pathways through which ICP disrupts lipid metabolism remain intricate, with some evidence pointing to anomalies in farnesoid X receptor (FXR) activity (40). Activating FXR leads to a suppression of endogenous bile acid synthesis while concurrently decreasing levels of triglycerides, total cholesterol, and glucose in the plasma (41). Increased levels of the 3β-sulfated progesterone metabolite epiallopregnanolone sulfate in ICP pregnancies antagonized the FXR (42, 43). Hence, the diminished activity of FXR could play a role in pregnancies with ICP, potentially affecting maternal metabolic processes (44). Although the mechanism is not yet clear, even the detection and intervention of dyslipidemia has important clinical value for the treatment of the disease itself and related complications in patients with ICP. In addition, it has been shown that gut microbiota may also play a role in lipid metabolism during ICP pregnancies (45). Although high bile acid levels have been implicated in cardiovascular toxicity, our MR analysis did not find substantial evidence to support the mediating role of liver function markers, including bile acids, in the association between ICP and CVD.

Interestingly, this analysis revealed a mediating role of HTN in the relationship between ICP and CAD. The connection between HTN and CAD is well-established and unequivocal. Chronic hypertension induces hemodynamic alterations that activate blood platelets, thereby accelerating the formation of atherosclerotic plaques (46). These plaques can lead to myocardial ischemia, hypoxia, or necrosis, culminating in CAD. Research by P. Oikonomou highlighted that patients with preeclampsia exhibit a pathophysiological profile akin to those observed in cardiovascular conditions, predisposing them to an increased risk of CAD (47). This evidence lends theoretical support to our study's findings.

This study explored the association between ICP and CVD at the genetic level. Because of the strong gene-phenotype association, the results of this study are limited to a causal relationship between a single disease (ICP) and CVD and cannot be extrapolated to other types of cholestasis. However, a review of the relevant literature shows that multiple causes of chronic cholestasis (biliary obstruction, PBC, and other diseases) seem to be associated with an increased risk of CVD and that abnormal lipid levels are involved in an important role (48–50). This suggests that a variety of cholestasis, including ICP, may modify the risk of CVD, but the causal relationship between them requires further subsequent validation.

This study boasts several notable strengths. Primarily, it is the inaugural MR analysis investigating the causal link between ICP and CVD, marking a significant advancement in this research domain. Additionally, we conducted a thorough examination of potential mediators within the causal pathway from ICP to CVD, enhancing the depth and breadth of our analysis. Nonetheless, the study is not without its limitations. A primary constraint is the exclusive manifestation of ICP in one sex, coupled with the absence of large-scale, sex-specific GWAS, necessitating the use of sex-combined population data. However, the selected SNPs for MR analysis exhibited no significant sex-based genetic effect differences, thereby minimally influencing the outcomes. Another concern is the potential for sample overlap between the lipid profiles and liver function markers employed in the mediation analysis. Furthermore, the reliance on data predominantly from European populations constrains the generalizability of our conclusions across diverse ethnic groups. Finally, despite the rigorous quality control adopted in this study to minimize the influence of confounding factors on the results, it is possible that the results may still be affected to some extent due to the extremely strong correlation between lipids and CVD itself.

In conclusion, it is important for individuals diagnosed with ICP to be made aware of their elevated risk for future CVD and the importance of monitoring lipid profiles for early intervention. Furthermore, there is a pressing need for additional research to elucidate the precise mechanisms through which ICP influences CVD risk.

## 5 Conclusion

In summary, this study is the first comprehensive MR analysis to explore the causal link between ICP and CVD using genome-wide data. Bidirectional MR analyses revealed that genetically predicted ICP is causally linked to an increased risk of CVD, with no evidence supporting a causal effect of genetically predicted CVD on ICP risk. Furthermore, MR-mediated analyses have substantiated both a direct causal impact of ICP on CVD risk and a notable indirect effect mediated through lipid profiles, specifically LDL, TC, and Apo-B. These results underscore the critical mediating role of lipids in the causal pathway from ICP to CVD.

## Data Availability

The original contributions presented in the study are included in the article/**Supplementary Material**, further inquiries can be directed to the corresponding authors.

## References

[1] Palmer KR Xiaohua L Mol BW. Management of intrahepatic cholestasis in pregnancy. Lancet. (2019) 393:853–4. 10.1016/S0140-6736(18)32323-7 30773279

[2] Saad AF Pacheco LD Chappell L Saade GR. Intrahepatic cholestasis of pregnancy: toward improving perinatal outcome. Reprod Sci Thousand Oaks Calif. (2022) 29:3100–5. 10.1007/s43032-021-00740-x 34524639

[3] Hobson S Gandhi S Sobel M. Intrahepatic cholestasis of pregnancy. CMAJ Can Med Assoc J J Assoc Medicale Can. (2022) 194:E1650. 10.1503/cmaj.220334 PMC9828981 36511865

[4] Williamson C Geenes V. Intrahepatic cholestasis of pregnancy. Obstet Gynecol. (2014) 124:120–33. 10.1097/AOG.0000000000000346 24901263

[5] Smith DD Rood KM. Intrahepatic cholestasis of pregnancy. Clin Obstet Gynecol. (2020) 63:134–51. 10.1097/GRF.0000000000000495 31764000

[6] Piechota J Jelski W. Intrahepatic cholestasis in pregnancy: review of the literature. J Clin Med. (2020) 9:1361. 10.3390/jcm9051361 32384779 PMC7290322

[7] Ovadia C Seed PT Sklavounos A Geenes V Di Ilio C Chambers J Association of adverse perinatal outcomes of intrahepatic cholestasis of pregnancy with biochemical markers: results of aggregate and individual patient data meta-analyses. Lancet Lond Engl. (2019) 393:899–909. 10.1016/S0140-6736(18)31877-4 PMC6396441 30773280

[8] Glantz A Marschall H-U Mattsson L-A. Intrahepatic cholestasis of pregnancy: relationships between bile acid levels and fetal complication rates. Hepatol Baltim Md. (2004) 40:467–74. 10.1002/hep.20336 15368452

[9] Ovadia C Sajous J Seed PT Patel K Williamson NJ Attilakos G Ursodeoxycholic acid in intrahepatic cholestasis of pregnancy: a systematic review and individual participant data meta-analysis. Lancet Gastroenterol Hepatol. (2021) 6:547–58. 10.1016/S2468-1253(21)00074-1 33915090 PMC8192305

[10] Gurung V Middleton P Milan SJ Hague W Thornton JG. Interventions for treating cholestasis in pregnancy. Cochrane Database Syst Rev. (2013) 2013(6):CD000493. 10.1002/14651858.CD000493.pub22 23794285 PMC7043272

[11] Dixon PH Williamson C. The pathophysiology of intrahepatic cholestasis of pregnancy. Clin Res Hepatol Gastroenterol. (2016) 40:141–53. 10.1016/j.clinre.2015.12.008 26823041

[12] Reyes H Báez ME González MC Hernández I Palma J Ribalta J Selenium, zinc and copper plasma levels in intrahepatic cholestasis of pregnancy, in normal pregnancies and in healthy individuals, in Chile. J Hepatol. (2000) 32:542–9. 10.1016/s0168-8278(00)80214-7 10782901

[13] Williamson C Hems LM Goulis DG Walker I Chambers J Donaldson O Clinical outcome in a series of cases of obstetric cholestasis identified via a patient support group. BJOG Int J Obstet Gynaecol. (2004) 111:676–81. 10.1111/j.1471-0528.2004.00167.x 15198757

[14] Xiao J Li Z Song Y Sun Y Shi H Chen D Molecular pathogenesis of intrahepatic cholestasis of pregnancy. Can J Gastroenterol Hepatol. (2021) 2021:6679322. 10.1155/2021/6679322 34195157 PMC8181114

[15] Goins EC Wein LE Watkins VY Campbell AIK Heine RP Hughes BL Maternal and neonatal outcomes in patients with hepatitis C and intrahepatic cholestasis of pregnancy: the sum of the parts. PLoS One. (2023) 18:e0293030. 10.1371/journal.pone.0293030 37851654 PMC10584137

[16] Terrault NA Williamson C. Pregnancy-associated liver diseases. Gastroenterology. (2022) 163:97–117.e1. 10.1053/j.gastro.2022.01.060 35276220

[17] Liu C Gao J Liu J Wang X He J Sun J Intrahepatic cholestasis of pregnancy is associated with an increased risk of gestational diabetes and preeclampsia. Ann Transl Med. (2020) 8:1574. 10.21037/atm-20-4879 33437773 PMC7791254

[18] Wu P Green M Myers JE. Hypertensive disorders of pregnancy. Br Med J. (2023) 381:e071653. 10.1136/bmj-2022-071653 37391211

[19] Fan X Zhou Q Zeng S Zhou J Peng Q Zhang M Impaired fetal myocardial deformation in intrahepatic cholestasis of pregnancy. J Ultrasound Med Off J Am Inst Ultrasound Med. (2014) 33:1171–7. 10.7863/ultra.33.7.1171 24958403

[20] Mor M Shmueli A Krispin E Bardin R Sneh-Arbib O Braun M Intrahepatic cholestasis of pregnancy as a risk factor for preeclampsia. Arch Gynecol Obstet. (2020) 301:655–64. 10.1007/s00404-020-05456-y 32034507

[21] Larsson SC Butterworth AS Burgess S. Mendelian randomization for cardiovascular diseases: principles and applications. Eur Heart J. (2023) 44:4913–24. 10.1093/eurheartj/ehad736 37935836 PMC10719501

[22] Skrivankova VW Richmond RC Woolf BAR Yarmolinsky J Davies NM Swanson SA Strengthening the reporting of observational studies in epidemiology using Mendelian randomization: the STROBE-MR statement. JAMA. (2021) 326:1614–21. 10.1001/jama.2021.18236 34698778

[23] Dixon PH Levine AP Cebola I Chan MMY Amin AS Aich A GWAS meta-analysis of intrahepatic cholestasis of pregnancy implicates multiple hepatic genes and regulatory elements. Nat Commun. (2022) 13:4840. 10.1038/s41467-022-29931-z 35977952 PMC9385867

[24] Rusk N. The UK biobank. Nat Methods. (2018) 15:1001. 10.1038/s41592-018-0245-2 30504882

[25] Kurki MI Karjalainen J Palta P Sipilä TP Kristiansson K Donner KM Finngen provides genetic insights from a well-phenotyped isolated population. Nature. (2023) 613:508–18. 10.1038/s41586-022-05473-8 36653562 PMC9849126

[26] Kintu C Soremekun O Kamiza AB Kalungi A Mayanja R Kalyesubula R The causal effects of lipid traits on kidney function in Africans: bidirectional and multivariable Mendelian-randomization study. EBioMedicine. (2023) 90:104537. 10.1016/j.ebiom.2023.104537 37001235 PMC10070509

[27] Burgess S Thompson SG. CRP CHD genetics collaboration. Avoiding bias from weak instruments in Mendelian randomization studies. Int J Epidemiol. (2011) 40:755–64. 10.1093/ije/dyr036 21414999

[28] Hartwig FP Davey Smith G Bowden J. Robust inference in summary data Mendelian randomization via the zero modal pleiotropy assumption. Int J Epidemiol. (2017) 46:1985–98. 10.1093/ije/dyx102 29040600 PMC5837715

[29] Chen X Kong J Pan J Huang K Zhou W Diao X Kidney damage causally affects the brain cortical structure: a Mendelian randomization study. EBioMedicine. (2021) 72:103592. 10.1016/j.ebiom.2021.103592 34619639 PMC8498227

[30] Ong J-S MacGregor S. Implementing MR-PRESSO and GCTA-GSMR for pleiotropy assessment in Mendelian randomization studies from a practitioner’s perspective. Genet Epidemiol. (2019) 43:609–16. 10.1002/gepi.22207 31045282 PMC6767464

[31] Sanderson E. Multivariable Mendelian randomization and mediation. Cold Spring Harb Perspect Med. (2021) 11:a038984. 10.1101/cshperspect.a038984 32341063 PMC7849347

[32] Wikström Shemer EA Stephansson O Thuresson M Thorsell M Ludvigsson JF Marschall H-U. Intrahepatic cholestasis of pregnancy and cancer, immune-mediated and cardiovascular diseases: a population-based cohort study. J Hepatol. (2015) 63:456–61. 10.1016/j.jhep.2015.03.010 25772037

[33] Hämäläinen S-T Turunen K Mattila KJ Sumanen M. Intrahepatic cholestasis of pregnancy and associated causes of death: a cohort study with follow-up of 27–46 years. BMC Womens Health. (2018) 18:1–5. 10.1186/s12905-018-0606-0 29914448 PMC6006795

[34] Erlinger S. Intrahepatic cholestasis of pregnancy: a risk factor for cancer, autoimmune and cardiovascular diseases? Clin Res Hepatol Gastroenterol. (2016) 40:139–40. 10.1016/j.clinre.2015.09.003 26500199

[35] Chen Y Zhang H Ning W Chen Y Wen C. The impact of intrahepatic cholestasis on pregnancy outcomes: a retrospective cohort study. BMC Gastroenterol. (2023) 23:16. 10.1186/s12876-023-02652-3 36653757 PMC9847161

[36] Zhang Y Lan X Cai C Li R Gao Y Yang L Associations between maternal lipid profiles and pregnancy complications: a prospective population-based study. Am J Perinatol. (2021) 38:834–40. 10.1055/s-0039-3402724 31891957

[37] Hartley A Haskard D Khamis R. Oxidized LDL and anti-oxidized LDL antibodies in atherosclerosis—novel insights and future directions in diagnosis and therapy. Trends Cardiovasc Med. (2019) 29:22–6. 10.1016/j.tcm.2018.05.010 29934015

[38] Winklhofer-Roob BM Faustmann G Roob JM. Low-density lipoprotein oxidation biomarkers in human health and disease and effects of bioactive compounds. Free Radic Biol Med. (2017) 111:38–86. 10.1016/j.freeradbiomed.2017.04.345 28456641

[39] Packard CJ Demant T Stewart JP Bedford D Caslake MJ Schwertfeger G Apolipoprotein B metabolism and the distribution of VLDL and LDL subfractions. J Lipid Res. (2000) 41:305–18. 10.1016/S0022-2275(20)32065-4 10681415

[40] Ma K Saha PK Chan L Moore DD. Farnesoid X receptor is essential for normal glucose homeostasis. J Clin Invest. (2006) 116:1102–9. 10.1172/JCI25604 16557297 PMC1409738

[41] Chiang JYL Ferrell JM. Bile acid receptors FXR and TGR5 signaling in fatty liver diseases and therapy. Am J Physiol Gastrointest Liver Physiol. (2020) 318:G554–73. 10.1152/ajpgi.00223.2019 31984784 PMC7099488

[42] Keitel V Dröge C Häussinger D. Targeting FXR in cholestasis. Handb Exp Pharmacol. (2019) 256:299–324. 10.1007/164_2019_231 31201556

[43] Abu-Hayyeh S Papacleovoulou G Lövgren-Sandblom A Tahir M Oduwole O Jamaludin NA Intrahepatic cholestasis of pregnancy levels of sulfated progesterone metabolites inhibit farnesoid X receptor resulting in a cholestatic phenotype. Hepatol Baltim Md. (2013) 57:716–26. 10.1002/hep.26055 PMC3592994 22961653

[44] Menżyk T Bator M Derra A Kierach R Kukla M. The role of metabolic disorders in the pathogenesis of intrahepatic cholestasis of pregnancy. Clin Exp Hepatol. (2018) 4:217–23. 10.5114/ceh.2018.80122 30603668 PMC6311745

[45] Schoeler M Caesar R. Dietary lipids, gut microbiota and lipid metabolism. Rev Endocr Metab Disord. (2019) 20:461–72. 10.1007/s11154-019-09512-0 31707624 PMC6938793

[46] Metoki H Iwama N Hamada H Satoh M Murakami T Ishikuro M Hypertensive disorders of pregnancy: definition, management, and out-of-office blood pressure measurement. Hypertens Res Off J Jpn Soc Hypertens. (2022) 45:1298–309. 10.1038/s41440-022-00965-6 PMC9207424 35726086

[47] Oikonomou P Tsonis O Paxinos A Gkrozou F Korantzopoulos P Paschopoulos M. Preeclampsia and long-term coronary artery disease: how to minimize the odds? Eur J Obstet Gynecol Reprod Biol. (2020) 255:253–8. 10.1016/j.ejogrb.2020.10.061 33153771

[48] Black DD. Chronic cholestasis and dyslipidemia: what is the cardiovascular risk? J Pediatr. (2005) 146:306–7. 10.1016/j.jpeds.2004.11.027 15756207

[49] Longo M Crosignani A Battezzati PM Giussani CS Invernizzi P Zuin M Hyperlipidaemic state and cardiovascular risk in primary biliary cirrhosis. Gut. (2002) 51:265–9. 10.1136/gut.51.2.265 12117892 PMC1773333

[50] Moezi L Dehpour AR. Cardiovascular abnormalities in obstructive cholestasis: the possible mechanisms. Liver Int. (2013) 33:7–15. 10.1111/j.1478-3231.2012.02803.x 22520558

