# Corrigendum: Genetically predicted lipids mediate the association between intrahepatic cholestasis of pregnancy and cardiovascular disease

**DOI:** 10.3389/fcvm.2024.1439868

**Published:** 2024-07-18

**Authors:** Ji Cui, Qilong Zhai, Mengjie Chen, Zhu Yang

**Affiliations:** ^1^ Department of Obstetrics and Gynecology, The Second Affiliated Hospital of Chongqing Medical University, Chongqing, China; ^2^ Department of Hepatobiliary Surgery, The Second Affiliated Hospital of Chongqing Medical University, Chongqing, China

**Keywords:** intrahepatic cholestasis of pregnancy, lipid, cardiovascular disease, Mendelian randomization, GWAS

A Corrigendum on Genetically predicted lipids mediate the association between intrahepatic cholestasis of pregnancy and cardiovascular disease By Cui J, Zhai Q, Chen M, Yang Z. (2024). Front. Cardiovasc. Med. 11:1401010. doi: 10.3389/fcvm.2024.1401010

In the published article, there was an error in Figure 1 as published. The SNP screening threshold was incorrectly written as *P* < 1 × 10^−8^. The correct threshold is *P* < 5 × 10^−8^. The corrected Figure 1 and it's caption appears below.

**Figure 1:**
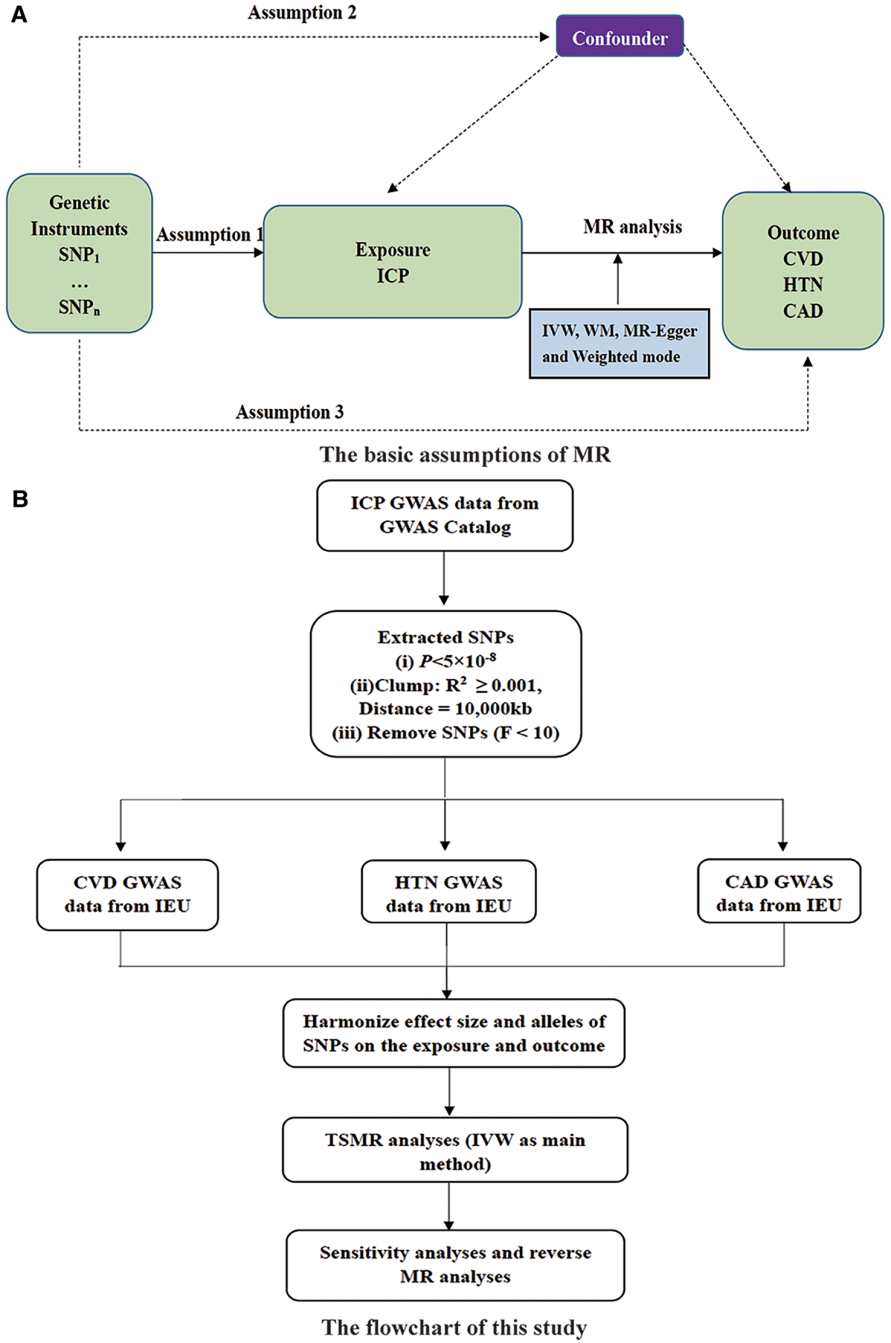
(**A**) The basic assumptions of MR and (**B**) study design. SNP, single nucleotide polymorphisms; ICP, intrahepatic cholestasis of pregnancy; IVW, inverse variance weighted; WM, weighted median; CVD, cardiovascular disease; HTN, hypertension; CAD, coronary artery disease; GWAS, genome-wide association studies; IEU, integrative epidemiology unit open GWAS project.

The authors apologize for this error and state that this does not change the scientific conclusions of the article in any way. The original article has been updated.

In the published article, there was an error. A prerequisite for the exclusion of outlier SNPs is missing before the description of the results of the sensitivity analyses.

A correction has been made to **Results**, 3.1 Causal effect between ICP and CVD (CAD, HTN) via TSMR. This sentence previously stated:

“The robustness of the results was assessed through Cochran's *Q* test, the MR-Egger intercept test and MR-PRESSO, as detailed in Supplementary Material Table S3.”

The corrected sentence appears below:

“After removing outlier SNP, the causal relationship between ICP and CVD still remained. The robustness of the results after removing outlier SNP was assessed through Cochran's Q test, the MR-Egger intercept test and MR-PRESSO, as detailed in Supplementary Material Table S3.”

The authors apologize for this error and state that this does not change the scientific conclusions of the article in any way. The original article has been updated.

In the published article, there was an error in Supplementary Table 3. The results of the sensitivity analyses in the original Supplementary Table 3 were incorrectly calculated due to incomplete data extraction and exclusion of outlier SNP values; the revised results are shown in the corrected Supplementary Table 3. The correct material statement appears below.

**Table:** 

Exposure-outcome	Q	Q *p*-value	MR-Egger interpreter	MR-Egger interpreter *p*-value	MR-PRESSO
ICP-CVD	9.525	0.146	0.019	0.377	0.244
ICP-HTN	15.110	0.035	0.000	0.931	0.068
ICP-CAD	0.001	0.977	–	–	–

The authors apologize for this error and state that this does not change the scientific conclusions of the article in any way. The original article has been updated.

